# The Influence of Intestinal Microbiota on BDNF Levels

**DOI:** 10.3390/nu16172891

**Published:** 2024-08-29

**Authors:** Marta Molska, Kinga Mruczyk, Angelika Cisek-Woźniak, Wojciech Prokopowicz, Patrycja Szydełko, Zuzanna Jakuszewska, Karolina Marzec, Martyna Trocholepsza

**Affiliations:** 1 Department of Dietetics, Faculty of Physical Culture in Gorzow Wlkp., Poznan University of Physical Education, Estkowskiego 13, 66-400 Gorzow Wielkopolski, Poland; k.mruczyk@awf-gorzow.edu.pl (K.M.); a.cisek@awf-gorzow.edu.pl (A.C.-W.); szydelko.patrycjaa@gmail.com (P.S.); zuzanna.jakuszewska@wp.pl (Z.J.); marzec.karolina2403@gmail.com (K.M.); martyna.trocholepsza@o2.pl (M.T.); 2 GSP Clinic Limited Liability Company, Kostrzyńska Street 12, 66-400 Gorzow Wielkopolski, Poland; recepcja@gspclinic.pl

**Keywords:** BDNF, neurotrophins, neurogenesis, microbiota, microbiome, probiotics, prebiotics, physical activity, dysbiosis

## Abstract

The regulation of neurogenesis, the complex process of producing and differentiating new brain tissue cells, is influenced by a complex interaction of internal and external factors. Over the past decade, extensive research has been conducted on neurotrophins and their key role in adult neurogenesis, as well as their impact on diseases such as depression. Among neurotrophins, the brain-derived neurotrophic factor (BDNF) has been the subject of comprehensive studies on adult neurogenesis, and scientific evidence supports its necessity for neurogenesis in the subventricular zone of the hippocampus. A novel area of research is the emerging role of gut microbiota as a significant contributor to neurogenesis and neurotrophin production. Studies have shown that reduced BDNF levels can lead to mood disorders, which are observed in intestinal dysbiosis, characterized by an imbalance in the composition and quantity of the intestinal microbiota. There is evidence in the literature that there is a link between brain function and gut microbiota. Physical activity, and especially the regularity and intensity of exercise, is important in relation to the level of BDNF and the intestinal microbiota. Probiotics, prebiotics and physical activity may have a positive effect on the intestinal microbiota, and therefore also on the level of the brain-derived neurotrophic factor.

## 1. Introduction

The brain consists of almost 100 billion neurons forming a dense network [1]. Each neuron can communicate with other neurons. The receiving neuron receives signals from the signal-sending neurons (presynaptic neurons) [2]. The efficient and integrated operation of the neural network allows us to receive stimuli from the surrounding world, think, and take appropriate actions [3]. 

Neurogenesis is the process by which new neurons are generated through the stem cells of the nervous system [4]. Neurotrophins are proteins that regulate the production, survival, proliferation, differentiation and death of neurons in the peripheral (PNS) and central (CNS) nervous systems [5]. The role of neurotrophins is to participate in the processes of neurogenesis (the differentiation, maturation, and survival of neurons) [6,7,8,9]. They are important for the control of cellular homeostasis, axon growth, dendritic branching, synaptogenesis, and cellular synaptic plasticity [6,7,8]. They can be produced by muscle cells, as well as transported retrogradely to the cell bodies of motor neurons, which are equipped with neurotrophin receptors [5]. During development, neurotrophins are essential for the survival of neurons, e.g., neurotrophin-3 (NT-3), and the brain-derived neurotrophic factor (BDNF) [10,11]. 

The brain-derived neurotrophic factor promotes brain cell survival through interactions with receptor tyrosine kinase B. It participates in synaptic plasticity of the central nervous system as well as the peripheral nervous system [12,13]. Publications indicate that the gastrointestinal microbiota, through changes in BDNF production, may have the ability to modulate behavior (normalize behavior) [14,15,16]. Scientists have been intensively studying microbiota for several years, i.e., microorganisms that colonize the human body. The set of microorganisms that inhabit a given habitat, together with their genes and the surrounding environmental conditions, is called the microbiome, and the term microbiota refers to the microorganisms that inhabit the microbiome [17,18]. The intestinal microbiota consists of trillions of microorganisms representing various species of bacteria, as well as fungi, viruses, protozoa, and archaea. The most abundant bacterial phyla are *Bacteroidetes* and *Firmicutes*, and the less common are *Proteobacteria* and *Actinobacteria*, *Fusobacteria,* and *Verrucomicrobia* [19,20,21].

Scientific publications indicate the important role of diverse intestinal microbiota in the proper functioning of the brain [22]. Its influence is noticeable in the synthesis and recognition of neurotransmitters, neurogenesis, myelination, brain development and the blood–brain barrier, as well as the maturation of the hypothalamic–pituitary–adrenal (HPA) axis [23,24,25,26]. 

Studies have shown that through the “gut-brain axis”, gut microbiota can influence human brain function. The “gut-brain axis” is a bidirectional communication between the gut and the brain that involves both neuronal and humoral pathways. It is composed of nerves from the nervous system associated with the intestines and the vagus nerve. In addition to direct connection via neurons, the microbiota can communicate with the central nervous system (CNS) via other mechanisms, i.e., hormonal and metabolic mechanisms, and those involving the immune system. Metabolites that are used for communication include short-chain fatty acids and neuroactive compounds. It is a bidirectional communication that influences processes such as neurogenesis, neurotransmission, and the regulation of the HPA axis [27,28,29,30,31,32,33].

The concept of the “microbiota-gut-brain axis” has been developed to describe the influence of human gut microbiota on homeostasis via the gut–brain axis [33]. Moreover, experimental results have shown that gut microbiota disorders are associated with the modulation of neuronal functions and brain metabolites [34,35]. The functioning of the microbiota depends on disturbed or normal intestinal physiology [36,37,38]. In turn, an imbalance in the intestinal microbiota, known as intestinal dysbiosis, may be caused by, for example, an inappropriate diet or too many pathogens. A weakened mental condition, which may be caused by long-term stress, may also contribute to a change in the intestinal microbiota composition [36,39,40,41]. 

The microbiota modulates some of the factors that exercise increases the levels of, e.g., the vascular endothelial factor, the brain-derived neurotrophic factor, and the insulin growth factor 1 (IGF-1) in the brain. Furthermore, increasing scientific evidence indicates that physical exercise (including low-intensity exercise and prolonged physical exercise) alters the composition of the gut microbiota and its associated metabolites [42,43,44,45,46,47,48,49]. Hence, the ability of exercise to shape the microbiome and pathways related to the brain and gut may be important for developing treatment strategies, e.g., for neurological disorders [42,43,44,45,46,47,48]. 

Prebiotics and probiotics influence the host microbiota and aim to improve the host’s health. Prebiotics are defined in the literature as non-digestible food ingredients that are selectively utilized by the gut microbiota. They have been shown to be a key modulator of the complex microbial community [50,51,52]. Prebiotic effects range from immune modulation, intestinal function, defense against pathogens, etc. [50,51,52]. In contrast, probiotics are defined as living organisms that can contribute to the health of the host and inhabit the gastrointestinal tract. *Lactobacillus* and *Bifidobacterium* are the most common microorganisms to be used as probiotics [50,53,54]. Probiotics can affect the development of host neurons, brain biochemistry, or a wide range of behavioral phenomena and are referred to as psychobiotics, especially those that taxonomically belong to *Lactobacilli* and *Bifidobacterium* [20,50,55]. 

Moreover, in light of the above information, there is a justified need for a review that, through the analysis of previously published scientific works, aims to clarify the relationship between gut microbiota and brain-derived neurotrophic factor (BDNF) levels. Such a review would also aim to identify factors that may influence BDNF levels through changes in the gut microbiota, including, but not limited to, probiotics, prebiotics, and physical activity.

## 2. The Digestive Tract Ecosystem

The digestive tract ecosystem is shaped from the moment of birth and changes throughout a person’s life [56,57,58]. Factors such as diet, stress, or infection may affect embryological development. Therefore, any disturbance in the mother’s condition during pregnancy may result in the appearance of atypical metabolites that may have a negative impact on the developing fetus [57,58,59]. 

There are indications in scientific publications that the microbiota begins to form already in the prenatal period. This is particularly important for stimulating the immature immune system as well as brain development in healthy newborns. This change in the prevailing dogma results from the examination of the fetal meconium and the mother’s placenta. Colonization of the duct before and after birth is a very significant event [57,60,61,62]. A critical point in the development of the nervous system is the perinatal period, while the composition of the microbiota at this stage is mainly *Proteobacteria* and *Actinobacteria* [57,60,61].

**Figure 1:**
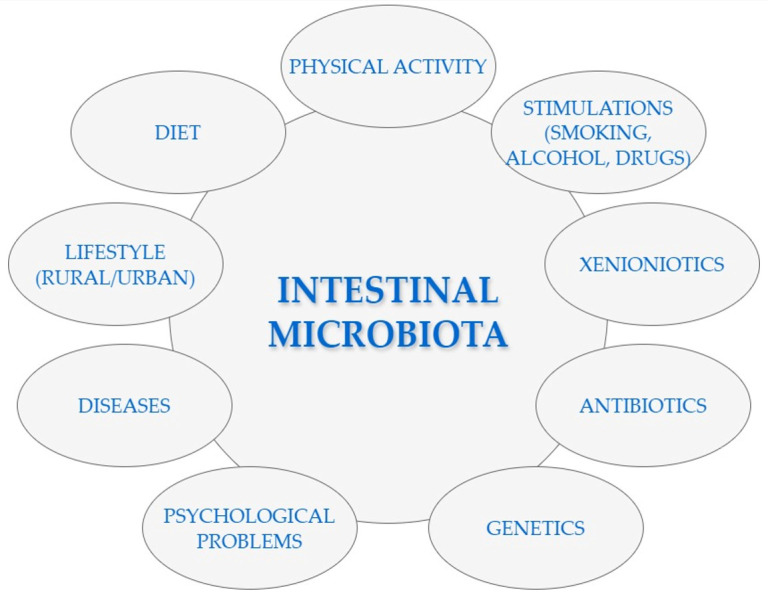
Selected factors that may affect the composition of the intestinal microbiota [14,63,64].

The gut microbiota composition depends on many factors, which are presented in Figure 1 [14,63,64]. 

The predominant phyla of gut microbes are *Firmicutes*, *Bacteroidetes*, *Actinobacteria*, *Proteobacteria*, *Fusobacteria*, and *Verrucomicrobia*. *Bacteroides* and *Firmicutes* are two phyla representing 90% of the gut microbiota [65,66]. The gut microbiota influences the host through immune, neural, neuroendocrine and metabolic pathways [39,66,67,68,69].

The functioning and development of the gut microbiota community early in life may cause long-term effects on the development of the central nervous system [70]. Microbial metabolites and synthesized neurotransmitters such as short-chain fatty acids (SCFAs), γ-aminobutyric acid, serotonin, norepinephrine, and dopamine are components of the gut–brain–microbiota axis [30,71,72,73,74]. These neurotransmitters have been shown to potentially affect microglia activation [74,75]. Neurotransmitters synthesized by microbes can cross the intestinal mucosa layer and the blood–brain barrier, and mediate physiological events in the brain [76]. Serotonin is important for regulating behavior, mood, sleep, and many other functions in the gastrointestinal tract and the central nervous system [74,75,77].

SCFAs may act as molecules that induce microglial maturation as well as increase serotonin biosynthesis in the colon [74]. Therefore, it is likely that microbial components and metabolites or products may be viable targets for the treatment or prevention of neuropsychiatric disorders. Potential new treatments that result from microbiome research include prebiotics, probiotics, and microbiota transplantation [78,79,80]. 

## 3. Intestinal Microbiota and BDNF

Bercik et al. demonstrated that the gut microbiota influences behavior and brain chemistry independently of the autonomic nervous system, inflammation, or gastrointestinal-specific neurotransmitters [16]. The intestinal microbiota is considered to be the so-called a “second brain” that can regulate brain development and functioning. The central nervous system and intestinal microbiota exchange information through neural, immunological and endocrine pathways [16,22,81]. 

The brain-derived neurotrophic factor (BDNF) belongs to the family of neurotrophins, or polypeptide growth factors. It plays an important role in neurogenesis and neuroplasticity [82,83,84]. The level of BDNF is influenced by several factors, e.g., inflammation, exposure to excessive stress, and the aging process. In addition to the factors indicated, the intestinal microbiome has been shown to play an important role in controlling host BDNF levels [85]. 

The BDNF is an important regulator of the expression and regulation of gastrointestinal tight junction proteins [82]. The gut microbiome is important for the proper development of both the CNS and the HPA axis early in life. A diverse microbiome communicates with the CNS, and tighter HPA control occurs. This may promote neuronal and gastrointestinal growth by regulating the BDNF [86,87,88,89].

Germ-free rodents showed lower BDNF expression in regions of the hippocampus and cerebral cortex [86]. In a study by Bistoletti et al., the authors found reduced BDNF protein levels in the hippocampus of young microbiota-depleted mice but unchanged BDNF mRNA levels [90]. The reason may be the distribution of the BDNF protein away from the translation site via axonal transport. There is no scientific evidence yet whether there is a relationship between changes in the composition of the microbiota and increased axonal transport [91,92].

Published data indicate that antibiotic-induced intestinal dysbiosis during adolescence may influence the expression of the brain-derived neurotrophic factor in both the central nervous system and the enteric nervous system (ENS) [90,93,94]. It should be emphasized that it has various effects on the indicated nervous systems in later periods of life [90,91,92]. Increased intestinal permeability causes an influx of intestinal microbial components (e.g., lipopolysaccharides), and the resulting systemic inflammation may lead to neuroinflammation in the central nervous system [85,95].

The gut microbiota can synthesize and recognize a range of neurochemicals, including neurotransmitters, neuroactive short-chain fatty acids (SCFAs), secondary bile acids, and other biologically active small molecules [14,96,97]. Studies show that metabolites derived from the intestinal microbiota (e.g., short-chain fatty acids) are very important molecular mediators in the microbiome–gut–brain (MGB) axis, e.g., short-chain fatty acids (SCFAs) increase the production of growth factors supporting the hippocampus, i.e., the BDNF [85,98,99].

Butyrate is one candidate that may link the gut microbiota with the regulation of BDNF levels in the brain [85,99]. *Faecalibacterium* is a microorganism that produces butyrate in the intestines. Butyrate affects the maintenance of brain-derived neurotrophic factor levels and neurogenesis in the hippocampus, as well as improving depressive behavior [100]. Butyrate, after being absorbed in the colon, is used by colonocytes to produce energy. Some of the energy reaches the brain across the blood–brain barrier by passing through systemic circulation [85,101,102]. 

In animal studies, butyrate has been shown to accelerate BDNF expression in the hippocampus by inhibiting histone deacetylase [103]. Butyrate maintains chromatin relaxation and thus increases BDNF expression in the hippocampus [104]. Publications have shown that patients diagnosed with major depressive disorder have lower levels of butyrate-producing bacteria in their gut microbiome. A cohort study found a positive correlation between butyrate-producing *Faecalibacterium* and *Coprococcus* bacteria and higher quality of life scores [34,101,105,106,107,108]. These results indicate that the pathophysiology of major depressive disorder may be modulated by butyrate, which is derived from the gut microbiota by maintaining BDNF expression [86]. 

The microbiota can produce a variety of amines, which in their uncharged form can penetrate the intestinal–vascular barrier. These compounds may be formed directly from bacteria or indirectly as a result of the action of bacteria on dietary ingredients [109,110,111,112,113,114,115,116,117,118,119]. In rats, fecal microbiota transplantation had an antidepressant effect in the treatment of depression by increasing, among others, BDNF expression levels and serotonin [120]. We can distinguish 5-hydroxytryptamine (serotonin); it is a monoaminergic neuromodulator [14,121]. Approximately 90% of serotonin is produced and secreted by the enterochromaffin cells of the intestines, which are strongly influenced by the intestinal microbiota. Additionally, the gut microbiota has been found to influence serotonin levels in the hippocampus, possibly by altering peripheral tryptophan availability [114,122,123,124,125].

The BDNF is present in the intestines. It is found in the epithelial and enteroendocrine cells of the mucosa, intestinal blood vessels, and in the smooth muscles of the external muscle as well as enteric and glial neurons [126,127]. A number of peptides that originate from the gastrointestinal tract can influence the BDNF and thus indirectly influence behavior, for example, the pancreatic polypeptide, which acts on the hypothalamic appetite centers to promote BDNF expression in the ventromedial satiety center. The pancreatic polypeptide may influence the regulation of food intake. Al-Qudah et al. showed that neuropeptides, i.e., substance P and the pituitary adenylyl cyclase-activating peptide (PACAP), released from intestinal motor neurons innervating the longitudinal muscle layer, increased the expression and secretion of the BDNF from smooth muscle cells [126,128]. Some of the produced compounds, for example claudin-2 (tight junction protein), may affect the barrier’s permeability and, therefore, the absorption of other, less permeable microbial products [129].

Moreover, it is worth noting that the conducted research shows that diosgenin (a steroid saponin with a neuroprotective effect) has an antidepressant effect, which is related to the strengthening of neurotrophic functions and the inhibition of inflammatory and neuroendocrine activity by regulating intestinal microflora [130]. Diosgenin causes a strong correlation between gut microbiota composition and inflammation, HPA axis activity, or hippocampal neurotrophic function [130].

Fröhlich et al. showed that BDNF expression in the hippocampus, the medial prefrontal cortex, and the hypothalamus was significantly reduced in mice treated with antibiotics [131]. In the study by Kayyal et al., the authors examined how short-term antibiotic treatment of newborns would affect the gut microbiome and the HPA axis. Compared to the control group, treated mice showed a higher abundance of *Firmicutes* and reduced BDNF levels [132]. 

## 4. Probiotics, Intestinal Microbiota, and BDNF

Probiotics can influence the composition of the intestinal microbiota. Additionally, publications have shown that probiotics may be responsible for better cognitive performance and that they are associated with increased BDNF expression [133,134,135].

Probiotic supplementation improves cognitive functions and mental stress. A significantly increased BDNF level was observed at week 12 of supplementation in a group taking probiotic supplements. Moreover, the obtained results suggest that *Eubacterium* and *Clostridiales* in the intestines caused by probiotic supplementation is closely associated with an increase in the BDNF in serum, which improves brain functions [136]. 

Sudo et al. showed that BDNF levels are lower in the cortex and hippocampus of germfree mice compared to controls. The colonization of sterile mice with feces from specific pathogen-free (SPF) mice or the administration of probiotics resulted in the partial and complete normalization of brain-derived neurotrophic factor behavior and levels, respectively [86]. Bercik et al. administered *Bifidobacterium* bacteria to rats and observed an increase in BDNF levels in the hippocampus [16].

Neurotransmitters, BDNF, and hormones are associated with the response to behavioral stress. The results obtained by Ding et al. suggest that *Akkermansia muciniphila*, by regulating abnormal fluctuations in the concentrations of neurotransmitters, hormones and BDNF expression levels, may regulate and alleviate depressive behavior in mice induced by chronic immobilization stress. The study also noted that the treatment also regulated the gut microbiota [137].

Probiotics can inhibit the growth of pathogenic bacteria as well as modulate the immune response of the mucosa and the intestinal microbiota [138,139]. In a study by Liang et al., the authors observed that the probiotic *Lactobacillus helveticus* NS8 could improve cognitive dysfunction induced by chronic immobilization stress in rats. Increased BDNF mRNA expression was observed in the hippocampus compared to the control group [140]. In mice treated with 1 × 10^10^ CFU of *Lactobacillus pentosus* var. *plantarum* C29, a normalization of expression of the brain-derived neurotrophic factor and interleukin-10, a tumor necrosis factor, was observed [141]. In a study examining the regulation of fetal microbiota and neurodevelopmental processes using the probiotics *Lactobacillus salivarius* (LAC) and *Bifidobacterium bifidum* (BIF) in the prenatal period, it was observed that BDNF levels were observed to be higher in the control group and the lipopolysaccharide-induced inflammation group than in the probiotic treatment group [139]. 

Consumption of the *Bifidobacterium longum* subspecies *infantis* CCFM687 strain improved stress-induced depressive behavior, increased the number of butyrate-producing bacteria and BDNF levels, and modulated the HPA axis in mice [142].

In the hippocampus, brain-derived neurotrophic factor expression levels were negatively correlated with *Akkermansiaceae*, *Helicobacteriaceae*, *Enterobacteriaceae*, and *Sutterellaceae* populations, which were positively correlated with inflammatory cytokine expression levels. The probiotics that increased BDNF expression in SH-SY5Y cells were *Lactobacillus* casei HY2782 and *Bifidobacterium lactis* HY8002 [143]. 

The administration of *Lactobacillus reuteri* NK33 and *Bifidobacterium adolescentis* NK98 to mice increased the population of *Bacteroidetes*, *Firmicutes,* and *Actinobacteria*. Moreover, they induced BDNF expression in the hippocampus [144]. *Actinobacteria* play an important role in maintaining intestinal permeability, inhibiting inflammatory processes, cross-feeding with other butyrate-producing bacteria, as well as direct involvement in neural mechanisms [145]. 

Li et al. observed a significantly changed composition of the intestinal microbiota; the number of bacteria of the genera *Alistipes*, *Alloprevotella,* and *Lleibacterium* decreased in rats as a result of knocking-out the Sigma-1 receptor (Sig-1R). The results obtained by the authors suggest that Sig-1R knockout leads to intestinal dysbiosis [146]. Sig-1R may exert neuroprotective effects by promoting BDNF expression [147].

According to Agnihotri et al. *Bifidobacterium* and *Lactobacilaceae* are two families containing many probiotic strains and are among the taxa with the most positive correlations with BDNF levels and neurogenesis [91].

## 5. Prebiotics, Intestinal Microbiota, and BDNF

Prebiotics (galactoolisaccharides (GOS) and fructooligosaccharides (FOS)) are soluble fibers. Many beneficial effects on the immune system and intestines are brought by increasing the proportion of *Bifidobacteria* and *Lactobacilli* in the intestines with the use of prebiotics [148,149,150,151]. Additionally, dietary ingredients such as prebiotic fiber are known to influence brain chemistry through the gut–brain axis [98]. It is emphasized that the influence on brain function through the increased production of neurotrophic factors, as well as neuroimmune signaling, is caused by short-chain fatty acids (SCFAs). SCFAs are produced as a result of the excessive consumption of soluble fiber [98].

Hebert et al. observed that maternal prebiotic intake influenced offspring behavior, gut microbiome composition, and brain gene expression in mice [152]. Consumption of prebiotics galactooligosaccharides (GOS) and fructooligosaccharides (FOS) also seems to be important in depression. They work by modulating the composition and number of intestinal microbiota and thus may influence depressive disorders and anxiety [153]. 

Animal studies have shown that prebiotics reduce the secretion of cortisol (by regulating the HPA axis), which is responsible for anxiety, stress, and the risk of depression [154,155]. Prebiotics also increase the concentration of the BDNF, which can cause depressive behavior when its levels are low [151,155,156]. After taking prebiotics, an increase in BDNF levels was observed in the hippocampus of rats. Authors Savignac et al. indicated that this is consistent with a probiotic effect and may be due to an increased number of *Bifidobacteria* in the intestines [151].

In a study, Church et al. showed that in rats, a diet with pectin-based fiber did not affect butyric or propionic acid, but increased circulating acetic acid was noted in both female and male rats. In addition, a diet rich in dietary fiber in the hippocampus was shown to increase the brain-derived neutrophil factor and decrease interleukin-6 (IL-6), interleukin-1 beta (IL-1β), interferon gamma (IFNγ), and the tumor necrosis factor (TNF-alpha). The authors showed that hippocampal neuroinflammation was inversely correlated with increased levels of short-chain fatty acids (SCFAs). Acetic acid was noted to be a potent mediator of increased BDNF production [98].

A particularly interesting compound is lactoferrin, an iron-binding glycoprotein that can affect BDNF expression. Lactoferrin has a positive effect on the growth of some probiotic strains. It can also affect the growth and diversification of the intestinal microbiota [157]. In a study conducted on piglets given a high dose of lactoferrin, a significant increase in the level of mRNA encoding BDNF was found compared to piglets given a lower dose of lactoferrin, as well as compared to the control group [157,158].

## 6. Physical Activity, Intestinal Microbiota, and BDNF

According to Walsh et al., physical activity is defined as “any voluntary bodily movement produced by skeletal muscle that requires energy expenditure, and is one of the most potent lifestyle factors influencing BDNF levels in the body and brain” [159]. Data presented in the literature indicate that aerobic exercise increases BDNF concentration, while strength training in most cases did not show such an effect. The effect that will be obtained depends largely on the duration and intensity [159,160,161,162,163].

Both physical activity and oral administration of *Bifidobacterium* can increase BDNF expression, promote neuronal survival, regeneration, and differentiation, enhance neuroprotection, improve nutrient delivery to the nervous system, and alleviate psychological stress [48,164,165]. The microbiota–gut–brain axis is activated when physical activity exceeds 60% of the maximum oxygen uptake (VO_2max_) or during long-term exercise training and disturbs the intestinal microbiota [63].

A randomized study involving 120 elderly people showed that aerobic training increases the anterior hippocampus’s size, consequently improving spatial memory. Increased serum brain-derived neurotrophic factor concentration is associated with increased hippocampal volume [166]. Macias et al. showed that seven days of moderate locomotor exercise contributed to increased BDNF protein expression in lumbar spinal cord neurons of rats [167].

Publications also indicate that physical exercise (including aerobic exercise) may have a positive impact on the activity of the intestinal microbiota and its diversity, which translates into beneficial health effects [45,168]. Changes in the composition of commensal bacteria have been associated with several neurological diseases. Authors Gaitan et al. found that aerobic exercise increased phenylalanine and alanine catabolites, but decreased serotonin levels [168]. Peripheral serotonin is produced by enterochromaffin cells in the intestines, and the intestinal microbiota can modulate the synthesis of this neurotransmitter through host cells. The decrease in plasma serotonin levels detected by the authors was correlated with a change in plasma BDNF levels [168,169,170].

Exhausting and irregular training (e.g., experienced by professional athletes) may contribute to intestinal microbiota dysbiosis. In a study in mice, strenuous exercise increased the growth of *Butyrivibrio* spp., *Oscillospira* spp., *Ruminococcus gnavus*, and *Coprococcus* spp., while decreasing the number of *Turicibacter* spp., as well as promoting intestinal inflammation. Prebiotics and probiotics have been proposed in addition to other dietary interventions to prevent intestinal dysbiosis or restore eubiosis and promote the recovery of athletes [63,171,172,173,174]. 

Animal studies have shown that exercise increases BDNF mRNA expression in several brain regions. Furthermore, exercise has been shown to significantly modulate inflammatory markers and also to affect the gut microbiota. The exact mechanisms by which physical activity induces the expression of the brain-derived neurotrophic factor are not yet understood. One theory suggests that the increase in BDNF levels by exercise may result from changes in epigenetic markers of brain-derived neurotrophic factor promoters [175,176,177,178,179].

Physical activity can improve, for example, *Akkermansia* bacteria (increased after aerobic exercise), *Firmicutes* (change in *Firmicutes*/*Bacteroidetes* ratio), short-chain fatty acids (increased concentration), and the gut–brain barrier. It can also affect the brain-derived neurotrophic factor, the hypothalamic–pituitary–adrenal axis (HPA axis), and the serotonin pathways of bidirectional gut–brain communication, thus contributing to reducing antagonistic psychological stress and maintaining body homeostasis [45,124,180,181,182,183,184].

## 7. Conclusions

To sum up, the BDNF is a protein that plays a key role in promoting the growth, development, and maintenance of neurons in the brain, taking part in various processes (e.g., neuroplasticity, neuronal survival, and synaptic modulation) [120].

The intestinal microbiota can synthesize and recognize a number of neurochemical substances, e.g., SCFAs. SCFAs, such as butyrate, contribute to increased BDNF expression, while intestinal dysbiosis may result in decreased BDNF expression, which may consequently affect synaptic plasticity and neuronal development [85].

Probiotics can affect both the composition of the gut microbiota and the level of the BDNF. Similarly, prebiotics can modulate the number and composition of the intestinal microbiota and increase the concentration of the BDNF, a low level of which may result in depressive behaviors.

Physical activity, especially aerobic training, positively affects both the intestinal microbiota and the increase in the expression and level of BDNF. Regularity and intensity of exercise are important.

Scientific publications complement existing evidence on the relationship between microbiota and brain processes. The gastrointestinal microbiota play a role in the elevation of brain BDNF levels. However, further research, especially clinical studies, is needed to better understand this interaction.

## Data Availability

The data used in this article are sourced from materials mentioned in the References section.

## References

[1] Herculano-Houzel S. (2012). The Remarkable, yet Not Extraordinary, Human Brain as a Scaled-up Primate Brain and Its Associated Cost. Proc. Natl. Acad. Sci. USA.

[2] (1997). The Principles of Nerve Cell Communication. Alcohol. Health Res. World.

[3] Parga N., Serrano-Fernández L., Falcó-Roget J. (2023). Emergent Computations in Trained Artificial Neural Networks and Real Brains. J. Inst..

[4] Cameron H.A., Glover L.R. (2015). Adult Neurogenesis: Beyond Learning and Memory. Annu. Rev. Psychol..

[5] Sahay A.S., Sundrani D.P., Joshi S.R. (2017). Neurotrophins: Role in Placental Growth and Development. Vitam. Horm..

[6] Notaras M., van den Buuse M. (2019). Brain-Derived Neurotrophic Factor (BDNF): Novel Insights into Regulation and Genetic Variation. Neuroscientist.

[7] Greenberg M.E., Xu B., Lu B., Hempstead B.L. (2009). New Insights in the Biology of BDNF Synthesis and Release: Implications in CNS Function. J. Neurosci..

[8] Kiyoshi C., Tedeschi A. (2020). Axon Growth and Synaptic Function: A Balancing Act for Axonal Regeneration and Neuronal Circuit Formation in CNS Trauma and Disease. Dev. Neurobiol..

[9] Huang E.J., Reichardt L.F. (2001). Neurotrophins: Roles in Neuronal Development and Function. Annu. Rev. Neurosci..

[10] Davis-López De Carrizosa M.A., Morado-Díaz C.J., Tena J.J., Benítez-Temiño B., Pecero M.L., Morcuende S.R., De La Cruz R.R., Pastor A.M. (2009). Complementary Actions of BDNF and Neurotrophin-3 on the Firing Patterns and Synaptic Composition of Motoneurons. J. Neurosci..

[11] Vidal P.P., Cullen K., Curthoys I.S., Du Lac S., Holstein G., Idoux E., Lysakowski A., Peusner K., Sans A., Smith P., Paxinos G. (2015). Chapter 28—The Vestibular System. The Rat Nervous System.

[12] Bathina S., Das U.N. (2015). Brain-Derived Neurotrophic Factor and Its Clinical Implications. Arch. Med. Sci..

[13] Matin S., Dadkhah M. (2024). BDNF/CREB Signaling Pathway Contribution in Depression Pathogenesis: A Survey on the Non-Pharmacological Therapeutic Opportunities for Gut Microbiota Dysbiosis. Brain Res. Bull..

[14] Maqsood R., Stone T.W. (2016). The Gut-Brain Axis, BDNF, NMDA and CNS Disorders. Neurochem. Res..

[15] González-Arancibia C., Urrutia-Piñones J., Illanes-González J., Martinez-Pinto J., Sotomayor-Zárate R., Julio-Pieper M., Bravo J.A. (2019). Do Your Gut Microbes Affect Your Brain Dopamine?. Psychopharmacology.

[16] Bercik P., Denou E., Collins J., Jackson W., Lu J., Jury J., Deng Y., Blennerhassett P., Macri J., McCoy K.D. (2011). The Intestinal Microbiota Affect Central Levels of Brain-Derived Neurotropic Factor and Behavior in Mice. Gastroenterology.

[17] Żakowicz J., Bramorska A., Zarzycka W., Kovbasiuk A., Kuć K., Brzezicka A. (2020). Wpływ mikrobiomu jelitowego na mózg i psychikę [The impact of the intestinal microbiome on the brain and psyche]. Kosmos.

[18] Berg G., Rybakova D., Fischer D., Cernava T., Vergès M.-C.C., Charles T., Chen X., Cocolin L., Eversole K., Corral G.H. (2020). Microbiome Definition Re-Visited: Old Concepts and New Challenges. Microbiome.

[19] Lankelma J.M., Nieuwdorp M., de Vos W.M., Wiersinga W.J. (2015). The Gut Microbiota in Internal Medicine: Implications for Health and Disease. Neth. J. Med..

[20] Umbrello G., Esposito S. (2016). Microbiota and Neurologic Diseases: Potential Effects of Probiotics. J. Transl. Med..

[21] Sun J., Chang E.B. (2014). Exploring Gut Microbes in Human Health and Disease: Pushing the Envelope. Genes Dis..

[22] Cryan J.F., Dinan T.G. (2012). Mind-Altering Microorganisms: The Impact of the Gut Microbiota on Brain and Behaviour. Nat. Rev. Neurosci..

[23] Kim G.-H., Shim J.-O. (2022). Gut Microbiota Affects Brain Development and Behavior. Clin. Exp. Pediatr..

[24] Lynch C.M., Nagpal J., Clarke G., Cryan J.F. (2021). Wrapping Things Up: Recent Developments in Understanding the Role of the Microbiome in Regulating Myelination. Curr. Opin. Physiol..

[25] Sudo N., Hyland N., Stanton C. (2016). Chapter 13—The Hypothalamic-Pituitary-Adrenal Axis and Gut Microbiota: A Target for Dietary Intervention?. The Gut-Brain Axis.

[26] Mou Y., Du Y., Zhou L., Yue J., Hu X., Liu Y., Chen S., Lin X., Zhang G., Xiao H. (2022). Gut Microbiota Interact With the Brain Through Systemic Chronic Inflammation: Implications on Neuroinflammation, Neurodegeneration, and Aging. Front. Immunol..

[27] Dinan T.G., Cryan J.F. (2017). The Microbiome-Gut-Brain Axis in Health and Disease. Gastroenterol. Clin. N. Am..

[28] El Aidy S., Dinan T.G., Cryan J.F. (2014). Immune Modulation of the Brain-Gut-Microbe Axis. Front. Microbiol..

[29] Perez-Burgos A., Wang B., Mao Y.-K., Mistry B., McVey Neufeld K.-A., Bienenstock J., Kunze W. (2013). Psychoactive Bacteria Lactobacillus Rhamnosus (JB-1) Elicits Rapid Frequency Facilitation in Vagal Afferents. Am. J. Physiol. Gastrointest. Liver Physiol..

[30] Sherwin E., Dinan T.G., Cryan J.F. (2018). Recent Developments in Understanding the Role of the Gut Microbiota in Brain Health and Disease. Ann. N. Y. Acad. Sci..

[31] Liang S., Wu X., Hu X., Wang T., Jin F. (2018). Recognizing Depression from the Microbiota–Gut–Brain Axis. Int. J. Mol. Sci..

[32] Iannone L., Preda A., Blottiere H., Clarke G., Albani D., Belcastro V., Carotenuto M., Cattaneo A., Citraro R., Ferraris C. (2019). Microbiota-Gut Brain Axis Involvement in Neuropsychiatric Disorders. Expert Rev. Neurother..

[33] Cryan J.F., O’Mahony S.M. (2011). The Microbiome-Gut-Brain Axis: From Bowel to Behavior. Neurogastroenterol. Motil..

[34] Hao Z., Wang W., Guo R., Liu H. (2019). Faecalibacterium Prausnitzii (ATCC 27766) Has Preventive and Therapeutic Effects on Chronic Unpredictable Mild Stress-Induced Depression-like and Anxiety-like Behavior in Rats. Psychoneuroendocrinology.

[35] Erny D., Dokalis N., Mezö C., Castoldi A., Mossad O., Staszewski O., Frosch M., Villa M., Fuchs V., Mayer A. (2021). Microbiota-Derived Acetate Enables the Metabolic Fitness of the Brain Innate Immune System during Health and Disease. Cell Metab..

[36] Martin C.R., Osadchiy V., Kalani A., Mayer E.A. (2018). The Brain-Gut-Microbiome Axis. Cell Mol. Gastroenterol. Hepatol..

[37] Collins S.M., Bercik P. (2009). The Relationship Between Intestinal Microbiota and the Central Nervous System in Normal Gastrointestinal Function and Disease. Gastroenterology.

[38] Jandhyala S.M., Talukdar R., Subramanyam C., Vuyyuru H., Sasikala M., Reddy D.N. (2015). Role of the Normal Gut Microbiota. World J. Gastroenterol..

[39] Foster J.A., Rinaman L., Cryan J.F. (2017). Stress & the Gut-Brain Axis: Regulation by the Microbiome. Neurobiol. Stress.

[40] Bested A.C., Logan A.C., Selhub E.M. (2013). Intestinal Microbiota, Probiotics and Mental Health: From Metchnikoff to Modern Advances: Part III—Convergence toward Clinical Trials. Gut Pathog..

[41] Brown K., DeCoffe D., Molcan E., Gibson D.L. (2012). Diet-Induced Dysbiosis of the Intestinal Microbiota and the Effects on Immunity and Disease. Nutrients.

[42] Llorens-Martín M., Torres-Alemán I., Trejo J.L. (2009). Mechanisms Mediating Brain Plasticity: IGF1 and Adult Hippocampal Neurogenesis. Neuroscientist.

[43] Morland C., Andersson K.A., Haugen Ø.P., Hadzic A., Kleppa L., Gille A., Rinholm J.E., Palibrk V., Diget E.H., Kennedy L.H. (2017). Exercise Induces Cerebral VEGF and Angiogenesis via the Lactate Receptor HCAR1. Nat. Commun..

[44] Gomes da Silva S., Unsain N., Mascó D.H., Toscano-Silva M., de Amorim H.A., Silva Araújo B.H., Simões P.S.R., Naffah-Mazzacoratti M.d.G., Mortara R.A., Scorza F.A. (2012). Early Exercise Promotes Positive Hippocampal Plasticity and Improves Spatial Memory in the Adult Life of Rats. Hippocampus.

[45] Monda V., Villano I., Messina A., Valenzano A., Esposito T., Moscatelli F., Viggiano A., Cibelli G., Chieffi S., Monda M. (2017). Exercise Modifies the Gut Microbiota with Positive Health Effects. Oxid. Med. Cell Longev..

[46] Lai Z., Shan W., Li J., Min J., Zeng X., Zuo Z. (2021). Appropriate Exercise Level Attenuates Gut Dysbiosis and Valeric Acid Increase to Improve Neuroplasticity and Cognitive Function after Surgery in Mice. Mol. Psychiatry.

[47] Mitchell C.M., Davy B.M., Hulver M.W., Neilson A.P., Bennett B.J., Davy K.P. (2019). Does Exercise Alter Gut Microbial Composition? A Systematic Review. Med. Sci. Sports Exerc..

[48] Nicolas S., Dohm-Hansen S., Lavelle A., Bastiaanssen T.F.S., English J.A., Cryan J.F., Nolan Y.M. (2024). Exercise Mitigates a Gut Microbiota-Mediated Reduction in Adult Hippocampal Neurogenesis and Associated Behaviours in Rats. Transl. Psychiatry.

[49] Bermon S., Petriz B., Kajėnienė A., Prestes J., Castell L., Franco O.L. (2015). The Microbiota: An Exercise Immunology Perspective. Exerc. Immunol. Rev..

[50] Sanders M.E., Merenstein D.J., Reid G., Gibson G.R., Rastall R.A. (2019). Probiotics and Prebiotics in Intestinal Health and Disease: From Biology to the Clinic. Nat. Rev. Gastroenterol. Hepatol..

[51] Kong Q., Liu T., Xiao H. (2022). Editorial: Effects of Probiotics and Prebiotics on Gut Pathogens and Toxins. Front. Microbiol..

[52] Yoo S., Jung S.-C., Kwak K., Kim J.-S. (2024). The Role of Prebiotics in Modulating Gut Microbiota: Implications for Human Health. Int. J. Mol. Sci..

[53] Papizadeh M., Rohani M., Nahrevanian H., Javadi A., Pourshafie M.R. (2017). Probiotic Characters of *Bifidobacterium* and *Lactobacillus* Are a Result of the Ongoing Gene Acquisition and Genome Minimization Evolutionary Trends. Microb. Pathog..

[54] Maftei N.-M., Raileanu C.R., Balta A.A., Ambrose L., Boev M., Marin D.B., Lisa E.L. (2024). The Potential Impact of Probiotics on Human Health: An Update on Their Health-Promoting Properties. Microorganisms.

[55] Rudzki L., Szulc A. (2018). “Immune Gate” of Psychopathology—The Role of Gut Derived Immune Activation in Major Psychiatric Disorders. Front. Psychiatry.

[56] Colella M., Charitos I.A., Ballini A., Cafiero C., Topi S., Palmirotta R., Santacroce L. (2023). Microbiota Revolution: How Gut Microbes Regulate Our Lives. World J. Gastroenterol..

[57] Molska M., Reguła J. (2019). Potential Mechanisms of Probiotics Action in the Prevention and Treatment of Colorectal Cancer. Nutrients.

[58] Romano-Keeler J., Weitkamp J.-H. (2015). Maternal Influences on Fetal Microbial Colonization and Immune Development. Pediatr. Res..

[59] Borre Y.E., O’Keeffe G.W., Clarke G., Stanton C., Dinan T.G., Cryan J.F. (2014). Microbiota and Neurodevelopmental Windows: Implications for Brain Disorders. Trends Mol. Med..

[60] Satokari R., Grönroos T., Laitinen K., Salminen S., Isolauri E. (2009). Bifidobacterium and Lactobacillus DNA in the Human Placenta. Lett. Appl. Microbiol..

[61] Dinan T.G., Stilling R.M., Stanton C., Cryan J.F. (2015). Collective Unconscious: How Gut Microbes Shape Human Behavior. J. Psychiatr. Res..

[62] Walker R.W., Clemente J.C., Peter I., Loos R.J. (2017). The Prenatal Gut Microbiome: Are We Colonized with Bacteria in Utero?. Pediatr. Obes..

[63] Wegierska A.E., Charitos I.A., Topi S., Potenza M.A., Montagnani M., Santacroce L. (2022). The Connection Between Physical Exercise and Gut Microbiota: Implications for Competitive Sports Athletes. Sports Med..

[64] Hasan N., Yang H. (2019). Factors Affecting the Composition of the Gut Microbiota, and Its Modulation. PeerJ.

[65] Arumugam M., Raes J., Pelletier E., Le Paslier D., Yamada T., Mende D.R., Fernandes G.R., Tap J., Bruls T., Batto J.-M. (2011). Enterotypes of the Human Gut Microbiome. Nature.

[66] Rinninella E., Raoul P., Cintoni M., Franceschi F., Miggiano G.A.D., Gasbarrini A., Mele M.C. (2019). What Is the Healthy Gut Microbiota Composition? A Changing Ecosystem across Age, Environment, Diet, and Diseases. Microorganisms.

[67] Naseribafrouei A., Hestad K., Avershina E., Sekelja M., Linløkken A., Wilson R., Rudi K. (2014). Correlation between the Human Fecal Microbiota and Depression. Neurogastroenterol. Motil..

[68] Dinan T.G., Cryan J.F. (2017). Brain–Gut–Microbiota Axis—Mood, Metabolism and Behaviour. Nat. Rev. Gastroenterol. Hepatol..

[69] Socała K., Doboszewska U., Szopa A., Serefko A., Włodarczyk M., Zielińska A., Poleszek E., Fichna J., Wlaź P. (2021). The Role of Microbiota-Gut-Brain Axis in Neuropsychiatric and Neurological Disorders—ScienceDirect. Pharmacol. Res..

[70] Sarkar A., Yoo J.Y., Valeria Ozorio Dutra S., Morgan K.H., Groer M. (2021). The Association between Early-Life Gut Microbiota and Long-Term Health and Diseases. J. Clin. Med..

[71] Fung T.C., Olson C.A., Hsiao E.Y. (2017). Interactions between the Microbiota, Immune and Nervous Systems in Health and Disease. Nat. Neurosci..

[72] Calvani R., Picca A., Lo Monaco M.R., Landi F., Bernabei R., Marzetti E. (2018). Of Microbes and Minds: A Narrative Review on the Second Brain Aging. Front. Med..

[73] Chaudhry T.S., Senapati S.G., Gadam S., Mannam H.P.S.S., Voruganti H.V., Abbasi Z., Abhinav T., Challa A.B., Pallipamu N., Bheemisetty N. (2023). The Impact of Microbiota on the Gut–Brain Axis: Examining the Complex Interplay and Implications. J. Clin. Med..

[74] Silva Y.P., Bernardi A., Frozza R.L. (2020). The Role of Short-Chain Fatty Acids From Gut Microbiota in Gut-Brain Communication—PMC. Front. Endocrinol..

[75] Zou H., Li J., Zhou J., Yi X., Cao S. (2021). Effects of Norepinephrine on Microglial Neuroinflammation and Neuropathic Pain. Ibrain.

[76] Rea K., Dinan T.G., Cryan J.F. (2016). The Microbiome: A Key Regulator of Stress and Neuroinflammation—PMC. Neurobiol. Stress..

[77] Abdel-Haq R., Schlachetzki J.C.M., Glass C.K., Mazmanian S.K. (2019). Microbiome-Microglia Connections via the Gut-Brain Axis. J. Exp. Med..

[78] Kho Z.Y., Lal S.K. (2018). The Human Gut Microbiome—A Potential Controller of Wellness and Disease. Front. Microbiol..

[79] Yuan L., Li Y., Chen M., Xue L., Wang J., Ding Y., Gu Q., Zhang J., Zhao H., Xie X. (2024). Therapeutic Applications of Gut Microbes in Cardiometabolic Diseases: Current State and Perspectives. Appl. Microbiol. Biotechnol..

[80] Cheng R., Xu T., Zhang Y., Wang F., Zhao L., Jiang Y., He F. (2020). Lactobacillus Rhamnosus GG and Bifidobacterium Bifidum TMC3115 Can Affect Development of Hippocampal Neurons Cultured In Vitro in a Strain-Dependent Manner. Probiotics Antimicrob. Proteins.

[81] Franzosa E.A., Huang K., Meadow J.F., Gevers D., Lemon K.P., Bohannan B.J.M., Huttenhower C. (2015). Identifying Personal Microbiomes Using Metagenomic Codes. Proc. Natl. Acad. Sci. USA.

[82] Li C., Cai Y.-Y., Yan Z.-X. (2018). Brain-Derived Neurotrophic Factor Preserves Intestinal Mucosal Barrier Function and Alters Gut Microbiota in Mice. Kaohsiung J. Med. Sci..

[83] Rosas-Vargas H., Martínez-Ezquerro J.D., Bienvenu T. (2011). Brain-Derived Neurotrophic Factor, Food Intake Regulation, and Obesity. Arch. Med. Res..

[84] Green M.J., Matheson S.L., Shepherd A., Weickert C.S., Carr V.J. (2011). Brain-Derived Neurotrophic Factor Levels in Schizophrenia: A Systematic Review with Meta-Analysis. Mol. Psychiatry.

[85] Suda K., Matsuda K. (2022). How Microbes Affect Depression: Underlying Mechanisms via the Gut–Brain Axis and the Modulating Role of Probiotics. Int. J. Mol. Sci..

[86] Sudo N., Chida Y., Aiba Y., Sonoda J., Oyama N., Yu X.-N., Kubo C., Koga Y. (2004). Postnatal Microbial Colonization Programs the Hypothalamic-Pituitary-Adrenal System for Stress Response in Mice. J. Physiol..

[87] Bravo J.A., Julio-Pieper M., Forsythe P., Kunze W., Dinan T.G., Bienenstock J., Cryan J.F. (2012). Communication between Gastrointestinal Bacteria and the Nervous System. Curr. Opin. Pharmacol..

[88] Diaz Heijtz R., Wang S., Anuar F., Qian Y., Björkholm B., Samuelsson A., Hibberd M.L., Forssberg H., Pettersson S. (2011). Normal Gut Microbiota Modulates Brain Development and Behavior. Proc. Natl. Acad. Sci. USA.

[89] Von Boyen G.B.T., Reinshagen M., Steinkamp M., Adler G., Kirsch J. (2002). Enteric Nervous Plasticity and Development: Dependence on Neurotrophic Factors. J. Gastroenterol..

[90] Bistoletti M., Caputi V., Baranzini N., Marchesi N., Filpa V., Marsilio I., Cerantola S., Terova G., Baj A., Grimaldi A. (2019). Antibiotic Treatment-Induced Dysbiosis Differently Affects BDNF and TrkB Expression in the Brain and in the Gut of Juvenile Mice. PLoS ONE.

[91] Agnihotri N., Mohajeri M.H. (2022). Involvement of Intestinal Microbiota in Adult Neurogenesis and the Expression of Brain-Derived Neurotrophic Factor. Int. J. Mol. Sci..

[92] Conner J.M., Lauterborn J.C., Yan Q., Gall C.M., Varon S. (1997). Distribution of Brain-Derived Neurotrophic Factor (BDNF) Protein and mRNA in the Normal Adult Rat CNS: Evidence for Anterograde Axonal Transport. J. Neurosci..

[93] Diamanti T., Prete R., Battista N., Corsetti A., De Jaco A. (2022). Exposure to Antibiotics and Neurodevelopmental Disorders: Could Probiotics Modulate the Gut–Brain Axis?. Antibiotics.

[94] Champagne-Jorgensen K., Kunze W.A., Forsythe P., Bienenstock J., McVey Neufeld K.-A. (2019). Antibiotics and the Nervous System: More than Just the Microbes?. Brain Behav. Immun..

[95] Solanki R., Karande A., Ranganathan P. (2023). Emerging Role of Gut Microbiota Dysbiosis in Neuroinflammation and Neurodegeneration. Front. Neurol..

[96] Lyte M. (2013). Microbial Endocrinology in the Microbiome-Gut-Brain Axis: How Bacterial Production and Utilization of Neurochemicals Influence Behavior. PLoS Pathog..

[97] Lyte M. (2014). Microbial Endocrinology: Host-Microbiota Neuroendocrine Interactions Influencing Brain and Behavior. Gut Microbes.

[98] Church J.S., Bannish J.A.M., Adrian L.A., Rojas Martinez K., Henshaw A., Schwartzer J.J. (2023). Serum Short Chain Fatty Acids Mediate Hippocampal BDNF and Correlate with Decreasing Neuroinflammation Following High Pectin Fiber Diet in Mice. Front. Neurosci..

[99] Guo C., Huo Y.-J., Li Y., Han Y., Zhou D. (2022). Gut-Brain Axis: Focus on Gut Metabolites Short-Chain Fatty Acids. World J. Clin. Cases.

[100] Knudsen J.K., Bundgaard-Nielsen C., Hjerrild S., Nielsen R.E., Leutscher P., Sørensen S. (2021). Gut Microbiota Variations in Patients Diagnosed with Major Depressive Disorder-A Systematic Review. Brain Behav..

[101] Sun J., Ling Z., Wang F., Chen W., Li H., Jin J., Zhang H., Pang M., Yu J., Liu J. (2016). *Clostridium Butyricum* Pretreatment Attenuates Cerebral Ischemia/Reperfusion Injury in Mice via Anti-Oxidation and Anti-Apoptosis. Neurosci. Lett..

[102] Boets E., Gomand S.V., Deroover L., Preston T., Vermeulen K., De Preter V., Hamer H.M., Van den Mooter G., De Vuyst L., Courtin C.M. (2017). Systemic Availability and Metabolism of Colonic-Derived Short-Chain Fatty Acids in Healthy Subjects: A Stable Isotope Study. J. Physiol..

[103] Tian P., Zhu H., Qian X., Chen Y., Wang Z., Zhao J., Zhang H., Wang G., Chen W. (2021). Consumption of Butylated Starch Alleviates the Chronic Restraint Stress-Induced Neurobehavioral and Gut Barrier Deficits Through Reshaping the Gut Microbiota. Front. Immunol..

[104] Heyck M., Ibarra A. (2019). Microbiota and Memory: A Symbiotic Therapy to Counter Cognitive Decline?. Brain Circ..

[105] Valles-Colomer M., Falony G., Darzi Y., Tigchelaar E.F., Wang J., Tito R.Y., Schiweck C., Kurilshikov A., Joossens M., Wijmenga C. (2019). The Neuroactive Potential of the Human Gut Microbiota in Quality of Life and Depression. Nat. Microbiol..

[106] Horn J., Mayer D.E., Chen S., Mayer E.A. (2022). Role of Diet and Its Effects on the Gut Microbiome in the Pathophysiology of Mental Disorders. Transl. Psychiatry.

[107] Radjabzadeh D., Bosch J.A., Uitterlinden A.G., Zwinderman A.H., Ikram M.A., van Meurs J.B.J., Luik A.I., Nieuwdorp M., Lok A., van Duijn C.M. (2022). Gut Microbiome-Wide Association Study of Depressive Symptoms. Nat. Commun..

[108] Maes M., Vasupanrajit A., Jirakran K., Klomkliew P., Chanchaem P., Tunvirachaisakul C., Payungporn S. (2023). Exploration of the Gut Microbiome in Thai Patients with Major Depressive Disorder Shows a Specific Bacterial Profile with Depletion of the Ruminococcus Genus as a Putative Biomarker. Cells.

[109] Miller P.E., Haberlen S.A., Brown T.T., Margolick J.B., DiDonato J.A., Hazen S.L., Witt M.D., Kingsley L.A., Palella F.J.J., Budoff M. (2016). Brief Report: Intestinal Microbiota-Produced Trimethylamine-: N: -Oxide and Its Association With Coronary Stenosis and HIV Serostatus. JAIDS J. Acquir. Immune Defic. Syndr..

[110] Wang Z., Roberts A.B., Buffa J.A., Levison B.S., Zhu W., Org E., Gu X., Huang Y., Zamanian-Daryoush M., Culley M.K. (2015). Non-Lethal Inhibition of Gut Microbial Trimethylamine Production for the Treatment of Atherosclerosis. Cell.

[111] Kuka J., Liepinsh E., Makrecka-Kuka M., Liepins J., Cirule H., Gustina D., Loza E., Zharkova-Malkova O., Grinberga S., Pugovics O. (2014). Suppression of Intestinal Microbiota-Dependent Production of pro-Atherogenic Trimethylamine N-Oxide by Shifting L-Carnitine Microbial Degradation. Life Sci..

[112] Williams B.B., Van Benschoten A.H., Cimermancic P., Donia M.S., Zimmermann M., Taketani M., Ishihara A., Kashyap P.C., Fraser J.S., Fischbach M.A. (2014). Discovery and Characterization of Gut Microbiota Decarboxylases That Can Produce the Neurotransmitter Tryptamine. Cell Host Microbe.

[113] Asano Y., Hiramoto T., Nishino R., Aiba Y., Kimura T., Yoshihara K., Koga Y., Sudo N. (2012). Critical Role of Gut Microbiota in the Production of Biologically Active, Free Catecholamines in the Gut Lumen of Mice. Am. J. Physiol. Gastrointest. Liver Physiol..

[114] Reigstad C.S., Salmonson C.E., Rainey J.F., Szurszewski J.H., Linden D.R., Sonnenburg J.L., Farrugia G., Kashyap P.C. (2015). Gut Microbes Promote Colonic Serotonin Production through an Effect of Short-Chain Fatty Acids on Enterochromaffin Cells. FASEB J..

[115] Frankenfeld C.L., Atkinson C., Wähälä K., Lampe J.W. (2014). Obesity Prevalence in Relation to Gut Microbial Environments Capable of Producing Equol or O-Desmethylangolensin from the Isoflavone Daidzein. Eur. J. Clin. Nutr..

[116] Cani P.D., Lecourt E., Dewulf E.M., Sohet F.M., Pachikian B.D., Naslain D., De Backer F., Neyrinck A.M., Delzenne N.M. (2009). Gut Microbiota Fermentation of Prebiotics Increases Satietogenic and Incretin Gut Peptide Production with Consequences for Appetite Sensation and Glucose Response after a Meal. Am. J. Clin. Nutr..

[117] Takagaki A., Otani S., Nanjo F. (2011). Antioxidative Activity of Microbial Metabolites of (-)-Epigallocatechin Gallate Produced in Rat Intestines. Biosci. Biotechnol. Biochem..

[118] García-Villalba R., Beltrán D., Espín J.C., Selma M.V., Tomás-Barberán F.A. (2013). Time Course Production of Urolithins from Ellagic Acid by Human Gut Microbiota. J. Agric. Food Chem..

[119] Kibe R., Kurihara S., Sakai Y., Suzuki H., Ooga T., Sawaki E., Muramatsu K., Nakamura A., Yamashita A., Kitada Y. (2014). Upregulation of Colonic Luminal Polyamines Produced by Intestinal Microbiota Delays Senescence in Mice. Sci. Rep..

[120] Cai T., Zheng S.-P., Shi X., Yuan L.-Z., Hu H., Zhou B., Xiao S.-L., Wang F. (2022). Therapeutic Effect of Fecal Microbiota Transplantation on Chronic Unpredictable Mild Stress-Induced Depression. Front. Cell Infect. Microbiol..

[121] Homberg J.R., Molteni R., Calabrese F., Riva M.A. (2014). The Serotonin-BDNF Duo: Developmental Implications for the Vulnerability to Psychopathology. Neurosci. Biobehav. Rev..

[122] Yano J.M., Yu K., Donaldson G.P., Shastri G.G., Ann P., Ma L., Nagler C.R., Ismagilov R.F., Mazmanian S.K., Hsiao E.Y. (2015). Indigenous Bacteria from the Gut Microbiota Regulate Host Serotonin Biosynthesis. Cell.

[123] Zhai L., Huang C., Ning Z., Zhang Y., Zhuang M., Yang W., Wang X., Wang J., Zhang L., Xiao H. (2023). Ruminococcus Gnavus Plays a Pathogenic Role in Diarrhea-Predominant Irritable Bowel Syndrome by Increasing Serotonin Biosynthesis. Cell Host Microbe.

[124] Clarke G., Grenham S., Scully P., Fitzgerald P., Moloney R.D., Shanahan F., Dinan T.G., Cryan J.F. (2013). The Microbiome-Gut-Brain Axis during Early Life Regulates the Hippocampal Serotonergic System in a Sex-Dependent Manner. Mol. Psychiatry.

[125] Loh J.S., Mak W.Q., Tan L.K.S., Ng C.X., Chan H.H., Yeow S.H., Foo J.B., Ong Y.S., How C.W., Khaw K.Y. (2024). Microbiota–Gut–Brain Axis and Its Therapeutic Applications in Neurodegenerative Diseases. Signal Transduct. Target. Ther..

[126] Al-Qudah M., Alkahtani R., Akbarali H.I., Murthy K.S., Grider J.R. (2015). Stimulation of Synthesis and Release of Brain-Derived Neurotropic Factor from Intestinal Smooth Muscle Cells by Substance P and Pituitary Adenylate Cyclase-Activating Peptide. Neurogastroenterol. Motil..

[127] Sharkey K.A., Mawe G.M. (2023). The Enteric Nervous System. Physiol. Rev..

[128] Sainsbury A., Shi Y.-C., Zhang L., Aljanova A., Lin Z., Nguyen A.D., Herzog H., Lin S. (2010). Y4 Receptors and Pancreatic Polypeptide Regulate Food Intake via Hypothalamic Orexin and Brain-Derived Neurotropic Factor Dependent Pathways. Neuropeptides.

[129] Liu X., Yang G., Geng X.-R., Cao Y., Li N., Ma L., Chen S., Yang P.-C., Liu Z. (2013). Microbial Products Induce Claudin-2 to Compromise Gut Epithelial Barrier Function. PLoS ONE.

[130] Cui J.-J., Huang Z.-Y., Xie Y.-H., Wu J.-B., Xu G.-H., Li C.-F., Zhang M.-M., Yi L.-T. (2023). Gut Microbiota Mediated Inflammation, Neuroendocrine and Neurotrophic Functions Involved in the Antidepressant-like Effects of Diosgenin in Chronic Restraint Stress. J. Affect. Disord..

[131] Fröhlich E.E., Farzi A., Mayerhofer R., Reichmann F., Jačan A., Wagner B., Zinser E., Bordag N., Magnes C., Fröhlich E. (2016). Cognitive Impairment by Antibiotic-Induced Gut Dysbiosis: Analysis of Gut Microbiota-Brain Communication. Brain Behav. Immun..

[132] Kayyal M., Javkar T., Firoz Mian M., Binyamin D., Koren O., McVey Neufeld K.-A., Forsythe P. (2020). Sex Dependent Effects of Post-Natal Penicillin on Brain, Behavior and Immune Regulation Are Prevented by Concurrent Probiotic Treatment. Sci. Rep..

[133] Huang T.-T., Lai J.-B., Du Y.-L., Xu Y., Ruan L.-M., Hu S.-H. (2019). Current Understanding of Gut Microbiota in Mood Disorders: An Update of Human Studies. Front. Genet..

[134] Ghezzi L., Cantoni C., Pinget G.V., Zhou Y., Piccio L. (2021). Targeting the Gut to Treat Multiple Sclerosis. J. Clin. Investig..

[135] Sivamaruthi B.S., Prasanth M.I., Kesika P., Chaiyasut C. (2019). Probiotics in Human Mental Health and Diseases—A Minireview. Trop. J. Pharm. Res..

[136] Kim C.-S., Cha L., Sim M., Jung S., Chun W.Y., Baik H.W., Shin D.-M. (2021). Probiotic Supplementation Improves Cognitive Function and Mood with Changes in Gut Microbiota in Community-Dwelling Older Adults: A Randomized, Double-Blind, Placebo-Controlled, Multicenter Trial. J. Gerontol. A Biol. Sci. Med. Sci..

[137] Ding Y., Bu F., Chen T., Shi G., Yuan X., Feng Z., Duan Z., Wang R., Zhang S., Wang Q. (2021). A Next-Generation Probiotic: Akkermansia Muciniphila Ameliorates Chronic Stress-Induced Depressive-like Behavior in Mice by Regulating Gut Microbiota and Metabolites. Appl. Microbiol. Biotechnol..

[138] Wu X., Vallance B.A., Boyer L., Bergstrom K.S.B., Walker J., Madsen K., O’Kusky J.R., Buchan A.M., Jacobson K. (2008). Saccharomyces Boulardii Ameliorates Citrobacter Rodentium-Induced Colitis through Actions on Bacterial Virulence Factors. Am. J. Physiol.-Gastrointest. Liver Physiol..

[139] Kar F., Hacioglu C., Kar E., Donmez D.B., Kanbak G. (2022). Probiotics Ameliorates LPS Induced Neuroinflammation Injury on Aβ 1-42, APP, γ-β Secretase and BDNF Levels in Maternal Gut Microbiota and Fetal Neurodevelopment Processes. Metab. Brain Dis..

[140] Liang S., Wang T., Hu X., Luo J., Li W., Wu X., Duan Y., Jin F. (2015). Administration of Lactobacillus Helveticus NS8 Improves Behavioral, Cognitive, and Biochemical Aberrations Caused by Chronic Restraint Stress. Neuroscience.

[141] Woo J.-Y., Gu W., Kim K.-A., Jang S.-E., Han M.J., Kim D.-H. (2014). Lactobacillus Pentosus Var. Plantarum C29 Ameliorates Memory Impairment and Inflammaging in a D-Galactose-Induced Accelerated Aging Mouse Model. Anaerobe.

[142] Tian P., Zou R., Song L., Zhang X., Jiang B., Wang G., Lee Y., Zhao J., Zhang H., Chen W. (2019). Ingestion of Bifidobacterium Longum Subspecies Infantis Strain CCFM687 Regulated Emotional Behavior and the Central BDNF Pathway in Chronic Stress-Induced Depressive Mice through Reshaping the Gut Microbiota. Food Funct..

[143] Ma X., Shin Y.-J., Park H.-S., Jeong J.-W., Kim J.Y., Shim J.-J., Lee J.-L., Kim D.-H. (2023). Lactobacillus Casei and Its Supplement Alleviate Stress-Induced Depression and Anxiety in Mice by the Regulation of BDNF Expression and NF-κB Activation. Nutrients.

[144] Jang H.-M., Lee K.-E., Kim D.-H. (2019). The Preventive and Curative Effects of Lactobacillus Reuteri NK33 and Bifidobacterium Adolescentis NK98 on Immobilization Stress-Induced Anxiety/Depression and Colitis in Mice. Nutrients.

[145] Binda C., Lopetuso L.R., Rizzatti G., Gibiino G., Cennamo V., Gasbarrini A. (2018). Actinobacteria: A Relevant Minority for the Maintenance of Gut Homeostasis. Dig. Liver Dis..

[146] Li J.-H., Liu J.-L., Li X.-W., Liu Y., Yang J.-Z., Chen L.-J., Zhang K.-K., Xie X.-L., Wang Q. (2023). Gut Microbiota from Sigma-1 Receptor Knockout Mice Induces Depression-like Behaviors and Modulates the cAMP/CREB/BDNF Signaling Pathway. Front. Microbiol..

[147] Mysona B.A., Zhao J., Smith S., Bollinger K.E. (2018). Relationship between Sigma-1 Receptor and BDNF in the Visual System. Exp. Eye Res..

[148] Vulevic J., Drakoularakou A., Yaqoob P., Tzortzis G., Gibson G.R. (2008). Modulation of the Fecal Microflora Profile and Immune Function by a Novel Trans-Galactooligosaccharide Mixture (B-GOS) in Healthy Elderly Volunteers. Am. J. Clin. Nutr..

[149] Van Vlies N., Hogenkamp A., Thijssen S., Dingjan G.M., Knipping K., Garssen J., Knippels L.M.J. (2012). Effects of Short-Chain Galacto- and Long-Chain Fructo-Oligosaccharides on Systemic and Local Immune Status during Pregnancy. J. Reprod. Immunol..

[150] Drakoularakou A., Tzortzis G., Rastall R.A., Gibson G.R. (2010). A Double-Blind, Placebo-Controlled, Randomized Human Study Assessing the Capacity of a Novel Galacto-Oligosaccharide Mixture in Reducing Travellers’ Diarrhoea. Eur. J. Clin. Nutr..

[151] Savignac H.M., Corona G., Mills H., Chen L., Spencer J.P.E., Tzortzis G., Burnet P.W.J. (2013). Prebiotic Feeding Elevates Central Brain Derived Neurotrophic Factor, N-Methyl-D-Aspartate Receptor Subunits and D-Serine. Neurochem. Int..

[152] Hebert J.C., Radford-Smith D.E., Probert F., Ilott N., Chan K.W., Anthony D.C., Burnet P.W.J. (2021). Mom’s Diet Matters: Maternal Prebiotic Intake in Mice Reduces Anxiety and Alters Brain Gene Expression and the Fecal Microbiome in Offspring. Brain Behav. Immun..

[153] Paiva I.H.R., Duarte-Silva E., Peixoto C.A. (2020). The Role of Prebiotics in Cognition, Anxiety, and Depression. Eur. Neuropsychopharmacol..

[154] Dziurkowska E., Wesolowski M. (2021). Cortisol as a Biomarker of Mental Disorder Severity. J. Clin. Med..

[155] Schmidt K., Cowen P.J., Harmer C.J., Tzortzis G., Errington S., Burnet P.W.J. (2015). Prebiotic Intake Reduces the Waking Cortisol Response and Alters Emotional Bias in Healthy Volunteers. Psychopharmacology.

[156] Burokas A., Arboleya S., Moloney R.D., Peterson V.L., Murphy K., Clarke G., Stanton C., Dinan T.G., Cryan J.F. (2017). Targeting the Microbiota-Gut-Brain Axis: Prebiotics Have Anxiolytic and Antidepressant-like Effects and Reverse the Impact of Chronic Stress in Mice. Biol. Psychiatry.

[157] Vega-Bautista A., de la Garza M., Carrero J.C., Campos-Rodríguez R., Godínez-Victoria M., Drago-Serrano M.E. (2019). The Impact of Lactoferrin on the Growth of Intestinal Inhabitant Bacteria. Int. J. Mol. Sci..

[158] Yang C., Zhu X., Liu N., Chen Y., Gan H., Troy F.A., Wang B. (2014). Lactoferrin Up-Regulates Intestinal Gene Expression of Brain-Derived Neurotrophic Factors BDNF, UCHL1 and Alkaline Phosphatase Activity to Alleviate Early Weaning Diarrhea in Postnatal Piglets. J. Nutr. Biochem..

[159] Walsh E.I., Smith L., Northey J., Rattray B., Cherbuin N. (2020). Towards an Understanding of the Physical Activity-BDNF-Cognition Triumvirate: A Review of Associations and Dosage. Ageing Res. Rev..

[160] Schmolesky M.T., Webb D.L., Hansen R.A. (2013). The Effects of Aerobic Exercise Intensity and Duration on Levels of Brain-Derived Neurotrophic Factor in Healthy Men. J. Sports Sci. Med..

[161] Huang T., Larsen K.T., Ried-Larsen M., Møller N.C., Andersen L.B. (2014). The Effects of Physical Activity and Exercise on Brain-Derived Neurotrophic Factor in Healthy Humans: A Review. Scand. J. Med. Sci. Sports.

[162] Cassilhas R.C., Lee K.S., Fernandes J., Oliveira M.G.M., Tufik S., Meeusen R., de Mello M.T. (2012). Spatial Memory Is Improved by Aerobic and Resistance Exercise through Divergent Molecular Mechanisms. Neuroscience.

[163] Dinoff A., Herrmann N., Swardfager W., Lanctôt K.L. (2017). The Effect of Acute Exercise on Blood Concentrations of Brain-Derived Neurotrophic Factor in Healthy Adults: A Meta-Analysis. Eur. J. Neurosci..

[164] Smith F., Clark J.E., Overman B.L., Tozel C.C., Huang J.H., Rivier J.E.F., Blikslager A.T., Moeser A.J. (2010). Early Weaning Stress Impairs Development of Mucosal Barrier Function in the Porcine Intestine. Am. J. Physiol. Gastrointest. Liver Physiol..

[165] Neeper S.A., Gómez-Pinilla F., Choi J., Cotman C. (1995). Exercise and Brain Neurotrophins. Nature.

[166] Erickson K.I., Voss M.W., Prakash R.S., Basak C., Szabo A., Chaddock L., Kim J.S., Heo S., Alves H., White S.M. (2011). Exercise Training Increases Size of Hippocampus and Improves Memory. Proc. Natl. Acad. Sci. USA.

[167] Macias M., Dwornik A., Skup M., Czarkowska-Bauch J. (2005). Confocal Visualization of the Effect of Short-Term Locomotor Exercise on BDNF and TrkB Distribution in the Lumbar Spinal Cord of the Rat: The Enhancement of BDNF in Dendrites?. Acta Neurobiol. Exp..

[168] Gaitán J.M., Moon H.Y., Stremlau M., Dubal D.B., Cook D.B., Okonkwo O.C., van Praag H. (2021). Effects of Aerobic Exercise Training on Systemic Biomarkers and Cognition in Late Middle-Aged Adults at Risk for Alzheimer’s Disease. Front. Endocrinol..

[169] Wipfli B., Landers D., Nagoshi C., Ringenbach S. (2011). An Examination of Serotonin and Psychological Variables in the Relationship between Exercise and Mental Health. Scand. J. Med. Sci. Sports.

[170] Park S.-A., Son S.Y., Lee A.-Y., Park H.-G., Lee W.-L., Lee C.H. (2020). Metabolite Profiling Revealed That a Gardening Activity Program Improves Cognitive Ability Correlated with BDNF Levels and Serotonin Metabolism in the Elderly. Int. J. Environ. Res. Public Health.

[171] Pane M., Amoruso A., Deidda F., Graziano T., Allesina S., Mogna L. (2018). Gut Microbiota, Probiotics, and Sport: From Clinical Evidence to Agonistic Performance. J. Clin. Gastroenterol..

[172] Lamprecht M., Frauwallner A. (2012). Exercise, Intestinal Barrier Dysfunction and Probiotic Supplementation. Med. Sport. Sci..

[173] Lamprecht M., Bogner S., Schippinger G., Steinbauer K., Fankhauser F., Hallstroem S., Schuetz B., Greilberger J.F. (2012). Probiotic Supplementation Affects Markers of Intestinal Barrier, Oxidation, and Inflammation in Trained Men; a Randomized, Double-Blinded, Placebo-Controlled Trial. J. Int. Soc. Sports Nutr..

[174] Clark A., Mach N. (2016). Exercise-Induced Stress Behavior, Gut-Microbiota-Brain Axis and Diet: A Systematic Review for Athletes. J. Int. Soc. Sports Nutr..

[175] Cotman C.W., Berchtold N.C., Christie L.-A. (2007). Exercise Builds Brain Health: Key Roles of Growth Factor Cascades and Inflammation. Trends Neurosci..

[176] Guan J.-S., Haggarty S.J., Giacometti E., Dannenberg J.-H., Joseph N., Gao J., Nieland T.J.F., Zhou Y., Wang X., Mazitschek R. (2009). HDAC2 Negatively Regulates Memory Formation and Synaptic Plasticity. Nature.

[177] Koppel I., Timmusk T. (2013). Differential Regulation of Bdnf Expression in Cortical Neurons by Class-Selective Histone Deacetylase Inhibitors. Neuropharmacology.

[178] Martínez-Guardado I., Arboleya S., Grijota F.J., Kaliszewska A., Gueimonde M., Arias N. (2022). The Therapeutic Role of Exercise and Probiotics in Stressful Brain Conditions. Int. J. Mol. Sci..

[179] Kang P., Wang A.Z.-X. (2024). Microbiota–Gut–Brain Axis: The Mediator of Exercise and Brain Health. Psychoradiology.

[180] Clarke S.F., Murphy E.F., O’Sullivan O., Lucey A.J., Humphreys M., Hogan A., Hayes P., O’Reilly M., Jeffery I.B., Wood-Martin R. (2014). Exercise and Associated Dietary Extremes Impact on Gut Microbial Diversity. Gut..

[181] Hintikka J.E., Ahtiainen J.P., Permi P., Jalkanen S., Lehtonen M., Pekkala S. (2023). Aerobic Exercise Training and Gut Microbiome-Associated Metabolic Shifts in Women with Overweight: A Multi-Omic Study. Sci. Rep..

[182] Kang S.S., Jeraldo P.R., Kurti A., Miller M.E.B., Cook M.D., Whitlock K., Goldenfeld N., Woods J.A., White B.A., Chia N. (2014). Diet and Exercise Orthogonally Alter the Gut Microbiome and Reveal Independent Associations with Anxiety and Cognition. Mol. Neurodegener..

[183] Torquati L., Gajanand T., Cox E.R., Willis C.R.G., Zaugg J., Keating S.E., Coombes J.S. (2023). Effects of Exercise Intensity on Gut Microbiome Composition and Function in People with Type 2 Diabetes. Eur. J. Sport Sci..

[184] Min L., Ablitip A., Wang R., Luciana T., Wei M., Ma X. (2024). Effects of Exercise on Gut Microbiota of Adults: A Systematic Review and Meta-Analysis. Nutrients.

